# Loss of SMARCB1 promotes autophagy and facilitates tumour progression in chordoma by transcriptionally activating ATG5

**DOI:** 10.1111/cpr.13136

**Published:** 2021-10-20

**Authors:** Mingxuan Li, Yutao Shen, Yujia Xiong, Shuai Wang, Chuzhong Li, Jiwei Bai, Yazhuo Zhang

**Affiliations:** ^1^ Beijing Neurosurgical Institute Capital Medical University Beijing China; ^2^ Department of Neurosurgery Beijing Tiantan Hospital, Capital Medical University Beijing China; ^3^ China National Clinical Research Center for Neurological Diseases Beijing China; ^4^ Brain Tumor Center Beijing Institute for Brain Disorders Beijing China

## Abstract

**Objectives:**

SWI/SNF‐related matrix‐associated actin‐dependent regulator of chromatin subfamily B member 1 (SMARCB1) loss is associated with a poor prognosis in chordoma, while the mechanism remains largely unclear. Here, we aim to explore the function and regulatory mechanisms of SMARCB1 in chordoma.

**Materials and Methods:**

The effect of SMARCB1 on chordoma cells was investigated in vitro and in vivo. Chromatin immunoprecipitation (ChIP) sequencing was used to investigate the mechanisms of SMARCB1 in chordoma. The association between SMARCB1 and autophagy was validated by Western blot, immunofluorescence and transmission electron microscopy. In addition, the ATG5 expression in chordoma tissue was assessed using immunohistochemistry and correlated with patient survival.

**Results:**

SMARCB1 inhibited the malignant phenotype of chordoma cells in vitro and in vivo, supporting a tumour suppressor role of SMARCB1 in chordoma. ATG5‐mediated autophagy was identified as a potential downstream pathway of SMARCB1. Mechanistically, SMARCB1 bound directly to the ATG5 promoter and epigenetically inhibited its transcription, which decreased ATG5 expression and impaired autophagy. Additionally, autophagy inhibitor chloroquine had a potential anti‐cancer effect on chordoma cells in vitro. Moreover, high ATG5 expression was observed in recurrent chordoma patients, which independently correlated with adverse outcomes.

**Conclusions:**

Taken together, our results revealed that the SMARCB1/ATG5 axis is a promising therapeutic target for chordoma and autophagy inhibitors may be effective agents for chordoma treatment.

## Graphical abstract

SMARCB1 bound directly to the promoter region of ATG5 and epigenetically inhibited transcription of ATG5, which impaired autophagy. Our finding highlights that the SMARCB1/ATG5 axis is a promising therapeutic target for chordoma and autophagy inhibitors may be effective agents for chordoma treatment.

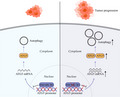

## 1 INTRODUCTION

Chordoma is a rare cancer with a poor prognosis, which is largely located in the spine and skull base, and is characterized by broad expression of Brachyury.^1^, ^2^ To date, surgical removal and adjuvant radiotherapy are recommended to prolong the survival of chordoma patients.^3^, ^4^ However, high recurrence and progression rates are frequently observed because of incomplete resection and resistance to radiotherapy.^1^ Additionally, conventional chemotherapeutic drugs have a limited effect on patient survival.^5^ Although clinical trials of potential target agents, which include imatinib, apatinib and a Brachyury vaccine, have been carried out recently,^6^, ^7^, ^8^ the results are unsatisfactory and the outcomes of chordoma patients remain dismal. Thus, identification of promising biomarkers and therapeutic targets in chordoma is urgent.

SMARCB1, a potential tumour suppressor gene located at chromosome 22q11.2, is a core component of the SWI/SNF complex that plays a prominent role in several cellular biological behaviours and the development of cancers.^9^, ^10^, ^11^ Numerous studies have revealed decreases in SMARCB1 expression or inactivation of SMARCB1 in various cancers, such as malignant rhabdoid tumours,^12^ familial schwannomatosis^13^ and epithelioid sarcomas.^14^ However, a recent study has indicated a potentially oncogenic role of SMARCB1 in liver cancer.^15^ In chordoma, our previous study and other studies showed that the low expression or loss of SMARCB1 correlate with dismal survival.^16^, ^17^, ^18^, ^19^ Moreover, recent studies proposed chordoma with SMARCB1 loss as a potential novel subset of chordoma, poorly differentiated chordoma, which displays aggressive behaviour and a dismal prognosis.^17^, ^18^, ^20^ However, the biological function and underlying mechanism of SMARCB1 in chordoma remain to be elucidated.

Autophagy is an evolutionarily conserved, cellular self‐protection process that is essential to maintain intracellular homeostasis via the degradation of cellular materials.^21^, ^22^ Increasing evidence demonstrates that autophagy participates in tumour progression and drug resistance of tumours, which suggests the therapeutic value of targeting autophagy.^23^, ^24^, ^25^ As an essential autophagy‐related gene (ATG), ATG5 plays a vital role in autophagosome formation and correlates with poor survival of several cancers.^26^, ^27^, ^28^ However, to date, the role of autophagy in chordoma remains largely unclear. Additionally, no previous study has investigated the expression, prognostic role or potential upstream regulators of ATG5 in chordoma.

Here, we examined the role and potential regulatory mechanism of SMARCB1 in chordoma. In knockdown and overexpression experiments, we found that SMARCB1 inhibited cell proliferation and invasion by regulating autophagy. Mechanistically, SMARCB1 bound directly to the promoter region of ATG5 and epigenetically inhibited transcription of ATG5, which impaired autophagy. Our results suggest the SMARCB1‐ATG5‐autophagy axis as a possible novel therapeutic target in chordoma.

## 2 MATERIALS AND METHODS

### 2.1 Cell culture

The human chordoma cell line UM‐Chor1 was purchased from the American Type Culture Collection (ATCC), and MUG‐Chor1 cells were kindly provided by the Chordoma Foundation. Cells were cultured in a mixture of IMDM (ATCC) and RPMI‐1640 (ATCC) culture media (4:1) supplemented with 10% foetal bovine serum and 1% antibiotics in a 5% CO2 incubator at 37°C.

### 2.2 Lentiviral vector, siRNA, and transfection

Lentiviral vectors for SMARCB1 knockdown and overexpression, and respective controls (named shNC, shSMARCB1, vector and SMARCB1 respectively) were acquired from Genechem (Shanghai, China). To establish stably transfected cell lines, 2 µg/ml puromycin was applied. Small interfering RNA (siRNA) against ATG5 (siATG5) and negative control (siNC) was obtained from RiboBio (Guangzhou, China). Chordoma cells were transfected using Lipofectamine 3000 (Invitrogen). Autophagy inhibitor chloroquine (CQ) was purchased from MedChemExpress. All sequences are detailed in Table S1.

### 2.3 RNA extraction and qRT‐PCR

Total RNA was isolated from chordoma cells using TRIzol reagent (Invitrogen), and the SuperScript III First Strand Synthesis System (Invitrogen, USA) was used to synthesize cDNA. qRT‐PCR was conducted on a QuantStudio 5 (Applied Biosystems) in triplicate. Target gene expression was normalized to GAPDH expression and shown as 2^−∆∆CT^. Primer sequences are provided in Table S2.

### 2.4 Western blot assay

Western blot was performed as described previously.^2^ The primary antibody against SMARCB1 was purchased from Bethyl (A301‐087A, 1:2500). A primary antibody against ATG5 was purchased from Proteintech (10181–2‐AP, 1:1000). A primary antibody against p62 was purchased from Cell Signaling Technology (39749, 1:1000). A primary antibody against LC3B was purchased from Novus (NB100‐2220, 1:500). An anti‐GAPDH antibody was purchased from ZSGB‐BIO.

### 2.5 Cell counting kit‐8 (CCK‐8) and colony formation assays

Cell viability was evaluated using CCK‐8 (Dojindo, Japan). Briefly, 2 × 10^3^ UM‐Chor1 cells per well and 6 × 10^3^ MUG‐Chor1 cells per well were seeded in a 96‐well plate. CCK‐8 was added at 0, 1, 2 and 3 days, and absorbance at 450 nm was then measured. For colony formation assays, 2 × 10^3^ transfected chordoma cells were seeded in a six‐well plate and incubated for 14 days. Colonies were then fixed with 4% paraformaldehyde, stained with crystal violet and counted.

### 2.6 Transwell migration and invasion assays

Briefly, 2 × 10^4^ UM‐Chor1 cells or 1 × 10^5^ MUG‐Chor1 cells were incubated in an 8.0‐µm Transwell chamber (Corning) with or without Matrigel. After 48 h, the migrated or invaded chordoma cells at the lower surface were fixed with paraformaldehyde and stained with crystal violet.

### 2.7 Chromatin immunoprecipitation (ChIP) sequencing and ChIP‐qPCR

UM‐Chor1 cells were applied to ChIP sequencing. Briefly, after cross‐linking by formaldehyde, the nuclear extract of the cells was collected and the chromatin was immunoprecipitated with an anti‐SMARCB1 antibody (ab12167, 10 μg; Abcam) or anti‐IgG antibody (8 μg; Millipore). High‐throughput DNA sequencing libraries were prepared by GeneCreate Biological Engineering Co., Ltd (Wuhan, China) using the VAHTS Universal DNA Library Prep Kit for Illumina V3 (Catalog No. ND607; Vazyme). The library products that corresponded to 200–500 bp were enriched, quantified and then sequenced on a Novaseq 6000 sequencer (Illumina). The data were analysed and mapped to the hg19 genome using STAR software with default parameters. MACS2 and bedtools software were used for peak calling and identification of different binding peaks. Function annotation of peak‐related genes was then conducted. ChIP‐qPCR was applied to confirm the ChIP sequencing results. The primers of ChIP‐qPCR are listed in Table S2.

### 2.8 Electrophoretic mobility shift assay

Reaction mixtures that contained biotin‐labelled ATG5 promoter probes (GeneCreate Biological Engineering Co., Ltd, Wuhan, China) and/or nuclear proteins of UM‐Chor1 cells were incubated with or without the anti‐SMARCB1 antibody (ab12167, Abcam) for 30 min according to the manufacturer's instructions. Then, the samples were subjected to 6% PAGE (80 V, 50 min), transferred to nylon membranes and finally visualized. The detailed probe information of the ATG5 promoter is shown in Table S3.

### 2.9 Immunofluorescence

A total of 5 × 10^3^ UM‐Chor1 cells or 2 × 10^4^ MUG‐Chor1 cells were seeded on coverslips in a 24‐well plate. After 24 h, the cells were fixed with 4% paraformaldehyde, permeabilized using 0.5% Triton X‐100, blocked by 5% goat serum and then incubated with the anti‐LC3B antibody (NB100‐2220, 1:200, Novus), followed by a Goat anti‐Rabbit Alexa Fluor 594 secondary antibody (A‐11037, 1:500; Thermo Fisher Scientific). Cells were mounted using a fluorescence mounting medium with DAPI (ZSGB‐BIO) and finally visualized under a Zeiss microscope.

### 2.10 Transmission electron microscopy

After collection using a cell scraper and centrifugation at 193 *g* for 5 min, chordoma cells were fixed with 2% glutaraldehyde and 1% osmium tetroxide, dehydrated in graded ethanol solutions and followed by embedment in epikote. Then, 50 nm ultrathin sections were constructed, stained with uranyl acetate and lead citrate and finally visualized under a transmission electron microscope (H‐7650; Hitachi, Japan).

### 2.11 Tumour specimens, immunohistochemistry (IHC) and analysis

A total of 84 histopathologically diagnosed skull base chordoma specimens from Beijing Tiantan Hospital between January 2008 and November 2010 were applied to IHC analysis. Additionally, 10 paired primary and recurrent specimens were also collected. The clinical information (age, classified by 55 years; gender; tumour volume, classified by 20 cm^3^; blood supply, abundant (tumour resection surface with a tendency to bleed and is hard to aspirate clearly), poor (limited bleeding that is easy to aspirate clearly) and moderate (between poor and abundant); texture, classified as hard (tumours could hardly be excised without scissor or punch forceps), soft (tumours could be easily suctioned when using the aspirator) and moderate (between hard and soft); etc.) of the 84 patients was reviewed as our previous study reported and is detailed in Table S4.^4^, ^29^, ^30^ IHC was performed using the Leica Bond III automated system as we have described previously.^2^ Primary antibody against ATG5 (sc‐133158, 1:50; Santa Cruz Biotechnology) was used for IHC of chordoma specimens. The IHC score of ATG5 was evaluated under a Leica Aperio AT2 scanner and defined as the product of the score of positive tumour cells as a percentage (0, no positive tumour cells; 1+, less than 25% cells; 2+, 25%‐50% cells; 3+, >50% cells) × the score of the staining intensity (0, no staining; 1+, weak, light yellow; 2+, moderate, yellow‐brown; 3+, strong, brown).^31^, ^32^ Low ATG5 expression was defined as an IHC score of <4, and IHC scores of ≥4 were defined as high ATG5 expression.

### 2.12 Xenograft model

To establish the chordoma model, 5 × 10^6^ MUG‐Chor1 cells were suspended in a mixture of 100 μl PBS and 100 μl Matrigel and then subcutaneously injected into the left flank of BALB/c nude mice (5–6 weeks of age, female; Beijing Vital River Laboratory Animal Technology). Tumour size was determined using the following formula: volume = (length × width^2^)/2. After 6 weeks, the tumour xenografts were extracted, weighed and applied to IHC staining for ATG5 (sc‐133158, 1:200; Santa Cruz Biotechnology) and LC3B (NB100‐2220, 1:400; Novus) and haematoxylin‐eosin (HE) staining.

### 2.13 Statistical methods

Data are presented as the mean ± SD. Statistical analysis was performed with SPSS 19.0 (IBM Corporation, USA) and GraphPad Prism 7.0 (GraphPad, USA). The Student's *t* test, paired *t* test and one‐way ANOVA were used for statistical analyses between groups. The chi‐squared test was used to compare clinicopathological characteristics between ATG5 subgroups. The Kaplan‐Meier survival curve and Cox analysis were applied for survival analysis. Statistical significance was based on *p* < 0.05.

## 3 RESULTS

### 3.1 SMARCB1 regulates the proliferation, migration, and invasion of chordoma cells

**FIGURE 1:**
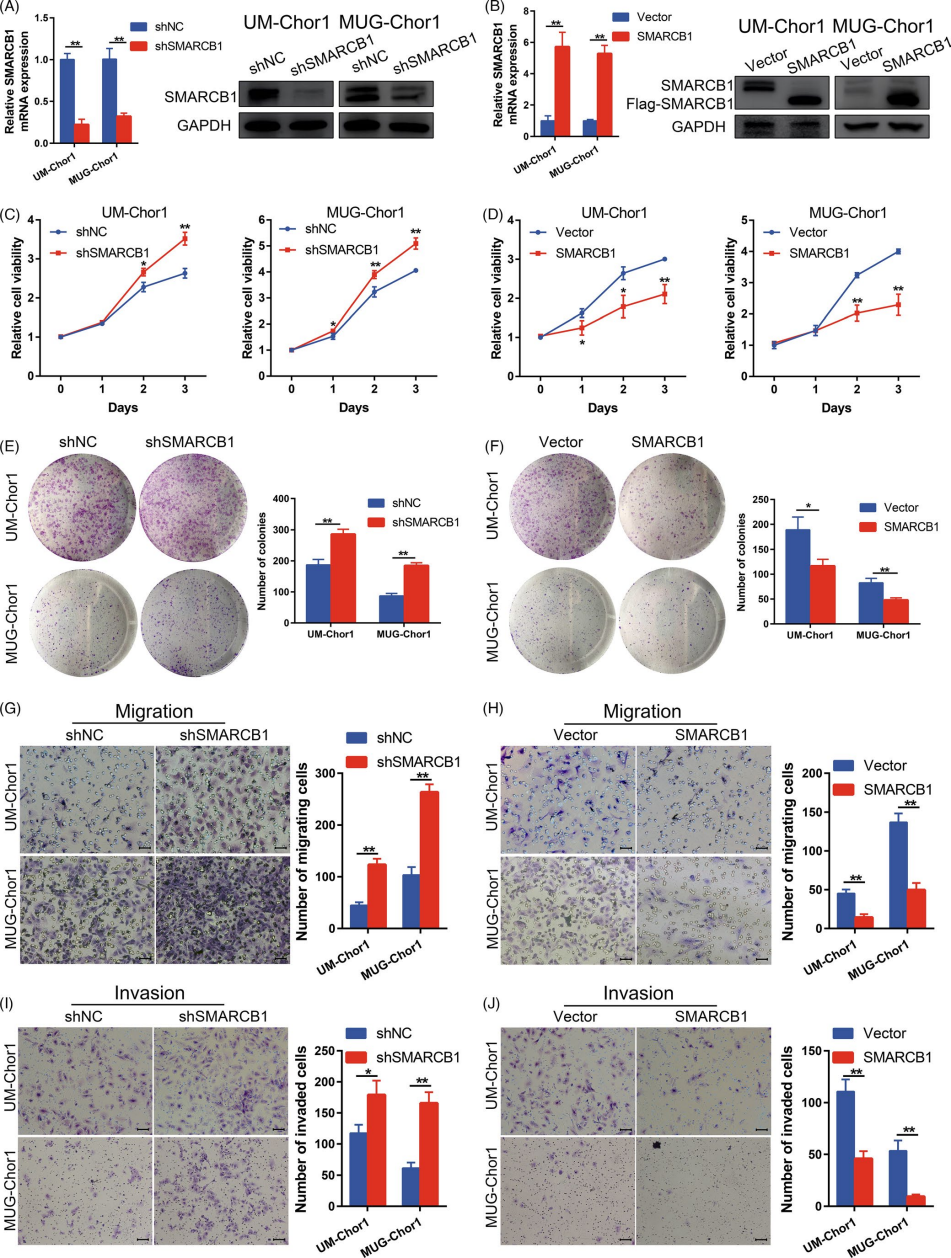
SMARCB1 played a tumour suppressor role in chordoma. (A and B) The mRNA and protein expression of SMARCB1 in UM‐Chor1 and MUG‐Chor1 cells with different transfection. (C and D) CCK‐8 assay of chordoma cells with SMARCB1 knockdown and overexpression. (E and F) Colony formation assay of chordoma cells with SMARCB1 knockdown and overexpression. (G–J) The migration and invasion ability of UM‐Chor1 and MUG‐Chor1 cells with different transfection. Scale bar, 50 µm for migration and 100 µm for invasion. Student's *t* test (A–B and E–J). One‐way ANOVA (C and D). *p < 0.05, **p < 0.01

We first established stably transfected SMARCB1 knockdown and overexpressing chordoma cells, and the efficiencies were checked by qRT‐PCR and Western blot (Figure 1A, B). Next, we analysed the effect of SMARCB1 on cell viability using CCK‐8 assay and observed increases in the viabilities of SMARCB1 knockdown UM‐Chor1 and MUG‐Chor1 cells (Figure 1C). Correspondingly, overexpression of SMARCB1 led to impaired cell viability (Figure 1D). Additionally, we found a similar in colony formation assay (Figure 1E, F). We also investigated the effect of SMARCB1 on the migration and invasion of chordoma cells. The results indicated that SMARCB1 knockdown significantly promoted the migration and invasion of chordoma cells, whereas SMARCB1 overexpression attenuated the migration and invasion of UM‐Chor1 and MUG‐Chor1 chordoma cells (Figure 1G–J). Together, these findings revealed that changes in SMARCB1 expression affected the malignant phenotype of chordoma cells.

### 3.2 **SMARCB1** **knockdown activates autophagy in chordoma cells**

**FIGURE 2:**
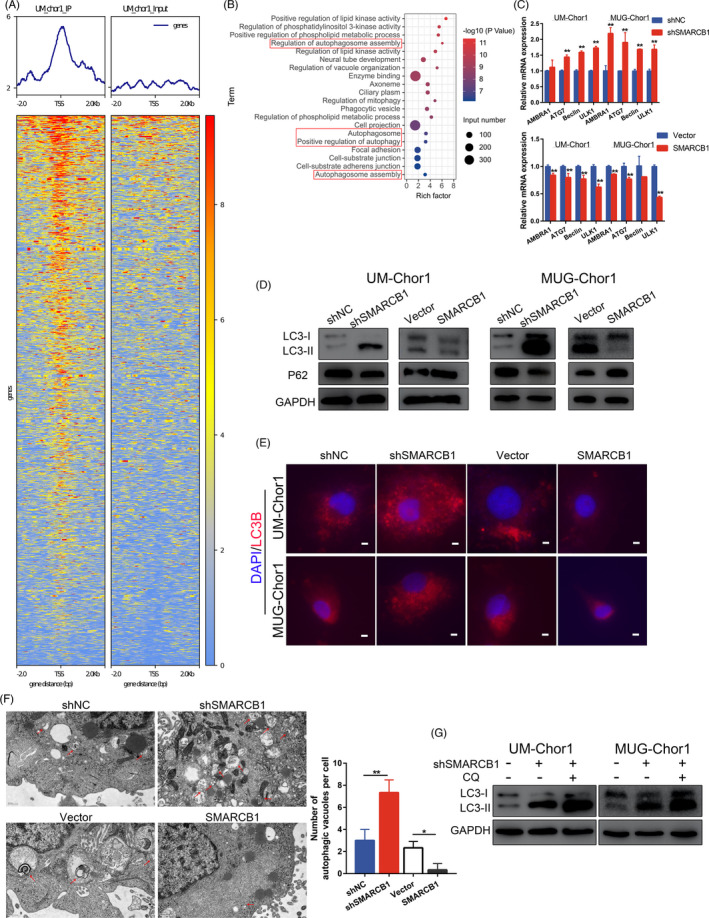
SMARCB1 regulated autophagy in chordoma cells. (A) ChIP sequencing of UM‐Chor1 cells suggested significant enrichment of SMARCB1‐associated peaks in TSS regions. (B) GO analysis of SMARCB1‐associated peaks identified enrichment of autophagy. (C) The mRNA expression of several autophagy‐related genes in UM‐Chor1 and MUG‐Chor1 cells. (D) The protein expression of LC3 and P62 in chordoma cells with different transfection. (E) Immunofluorescence images of LC3B and DAPI in chordoma cells under different transfection. Scale bar, 5 µm. (F) Transmission electron microscopy images of autophagic vacuoles in chordoma cells. Scale bar, 0.5 µm. (G) The protein expression of LC3 in chordoma cells with different treatments. Student's *t* test (C). One‐way ANOVA (F). *p < 0.05, **p < 0.01

We then asked the potential downstream molecules mediated by SMARCB1 in chordoma cells by ChIP sequencing of UM‐Chor1 cells incubated with or without an anti‐SMARCB1 antibody. We identified 6714 peaks, which corresponded to 2957 genes, in the SMARCB1 group compared with the input control (Figure S1A). Significant enrichment of SMARCB1‐associated peaks in the transcription start site (TSS) region was observed, which revealed the potential regulation of transcription by SMARCB1 (Figure 2A). Interestingly, Gene Ontology (GO) analysis of related genes identified several autophagy‐related terms that included regulation of autophagosome assembly, autophagosome and autophagosome assembly (Figures 2B, S1B–D). Thus, we speculated that SMARCB1 may function via autophagy in chordoma, and we then explored the ATGs in cells with changes in SMARCB1 expression. The mRNA expression of several ATGs was increased after SMARCB1 knockdown, which was decreased in cells that overexpressed SMARCB1 (Figure 2C). We also found changes in expression of two autophagy markers, LC3B and P62, in these cells (Figure 2D). Moreover, we validated the negative association between autophagy and SMARCB1 by immunofluorescence and observed decreased LC3B puncta in SMARCB1 knockdown cells and augmented LC3B puncta in SMARCB1‐overexpressing cells (Figure 2E). Transmission electron microscopy (TEM) analysis further revealed significant changes in autophagic vacuoles in SMARCB1 knockdown and overexpressing cells (Figure 2F). To further explore whether SMARCB1 knockdown promoted autophagy via enhanced autophagy flux or impaired autophagosome clearance, we treated SMARCB1 knockdown cells with lysosomal inhibitor CQ, which increased the LC3‐II expression in SMARCB1 knockdown UM‐Chor1 and MUG‐Chor1 cells (Figure 2G). Taken together, these findings suggested that SMARCB1 knockdown induced autophagic flux in chordoma cells.

### 3.3 SMARCB1 negatively regulates ATG5 expression by directly targeting the promoter of ATG5

**FIGURE 3:**
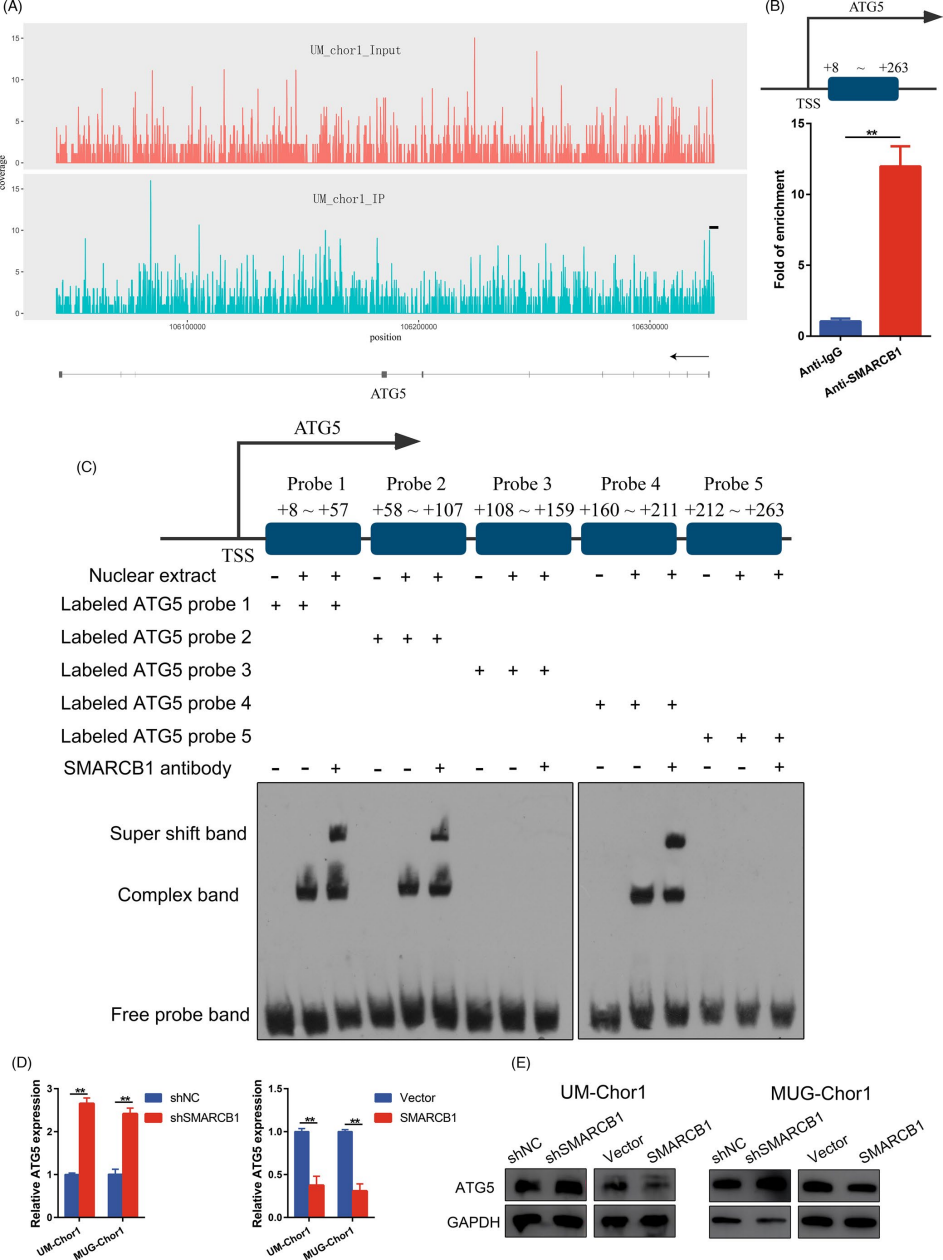
SMARCB1 regulated ATG5 expression by directly binding to the ATG5 promoter. (A) ChIP sequencing revealed the potential peak of SMARCB1 around the ATG5 promoter. (B) ChIP‐qPCR results of UM‐Chor1 cells confirmed the potential bind of SMARCB1. (C) Electrophoretic mobility shift assay of UM‐Chor1 cells showed probes 1, 2 and 4 of ATG5 promoter could form the supershift band with the nuclear extract and SMARCB1 antibody, further confirming the binding of SMARCB1 at ATG5 promoter. (D and E) The mRNA and protein expression of ATG5 in UM‐Chor1 and MUG‐Chor1 cells with different transfection. Student's *t* test (D). **p < 0.01

We next explored the potential autophagy‐related targets of SMARCB1. ChIP sequencing suggested potential enrichment of SMARCB1 in the promoter region of ATG5 (Figure 3A). The subsequent ChIP‐qPCR verified binding of SMARCB1 to the ATG5 promoter (+8 to +263 bp) (Figure 3B). To further clarify the detailed binding site, we designed five probes of the binding region and performed an electrophoretic mobility shift assay (EMSA). The results showed probes 1, 2 and 4 formed a supershift band with the nuclear extract and SMARCB1 antibody (Figure 3C). We also found augmentation of ATG5 expression in SMARCB1 knockdown cells and impaired ATG5 expression in SMARCB1‐overexpressing chordoma cells (Figure 3D and E).

### 3.4 **ATG5** **knockdown and an autophagy inhibitor reverse the effect of SMARCB1 on chordoma cells**

**FIGURE 4:**
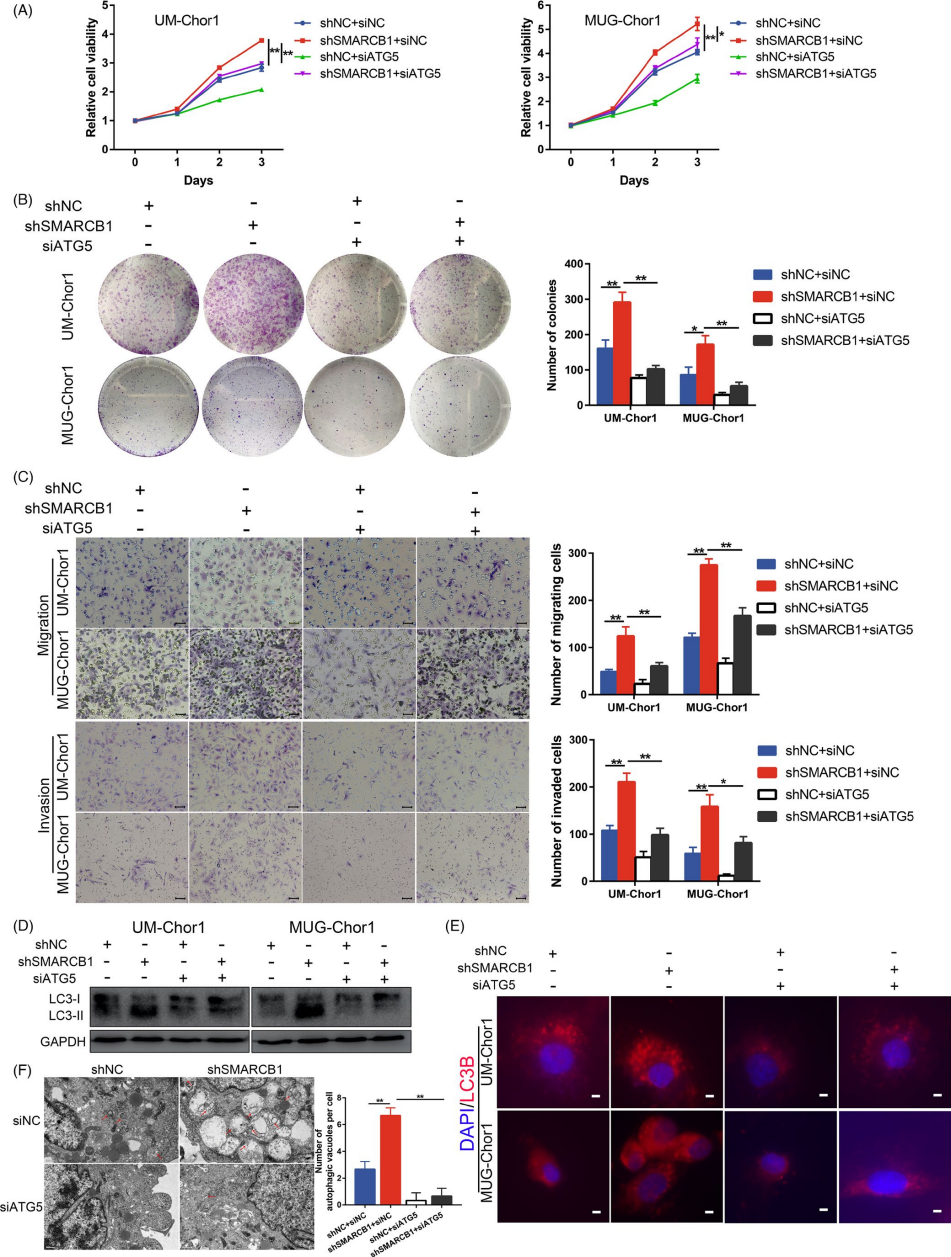
ATG5 knockdown reversed the SMARCB1 loss‐mediated malignant phenotype. (A) CCK‐8 assay of UM‐Chor1 and MUG‐Chor1 cells with or without ATG5 knockdown. (B) Colony formation assay of UM‐Chor1 and MUG‐Chor1 cells with or without ATG5 knockdown. (C) The migration and invasion of UM‐Chor1 and MUG‐Chor1 cells with or without ATG5 knockdown. Scale bar, 50 µm for migration and 100 µm for invasion. (D) The protein expression of LC3 in chordoma cells with or without ATG5 knockdown. (E) Immunofluorescence images of LC3B and DAPI in chordoma cells with or without ATG5 knockdown. Scale bar, 5 µm. (F) Transmission electron microscopy images of autophagic vacuoles in chordoma cells with or without ATG5 knockdown. Scale bar, 0.5 µm. One‐way ANOVA (A–C and F). *p < 0.05, **p < 0.01

To determine whether ATG5 participates in the tumour suppressor role of SMARCB1 in chordoma, we examined the effect of ATG5 knockdown in UM‐Chor1 and MUG‐Chor1 cells with SMARCB1 knockdown. The mRNA and protein expression of ATG5 were successfully inhibited by ATG5 siRNA (Figure S2A and B). The CCK‐8 assay revealed that the SMARCB1 knockdown‐mediated increase in proliferation was suppressed by ATG5 inhibition (Figure 4A). Additionally, the colony number was consistently decreased after ATG5 inhibition (Figure 4B). Transwell assays also identified suppressed migration and invasion of SMARCB1 knockdown cells treated with ATG5 siRNA (Figure 4C). Of note, the enhanced autophagy in SMARCB1 knockdown cells was impaired by ATG5 siRNA, as suggested by the attenuated LC3‐II level, decreased LC3B puncta and reduced autophagic vacuoles (Figure 4D–F).

**FIGURE 5:**
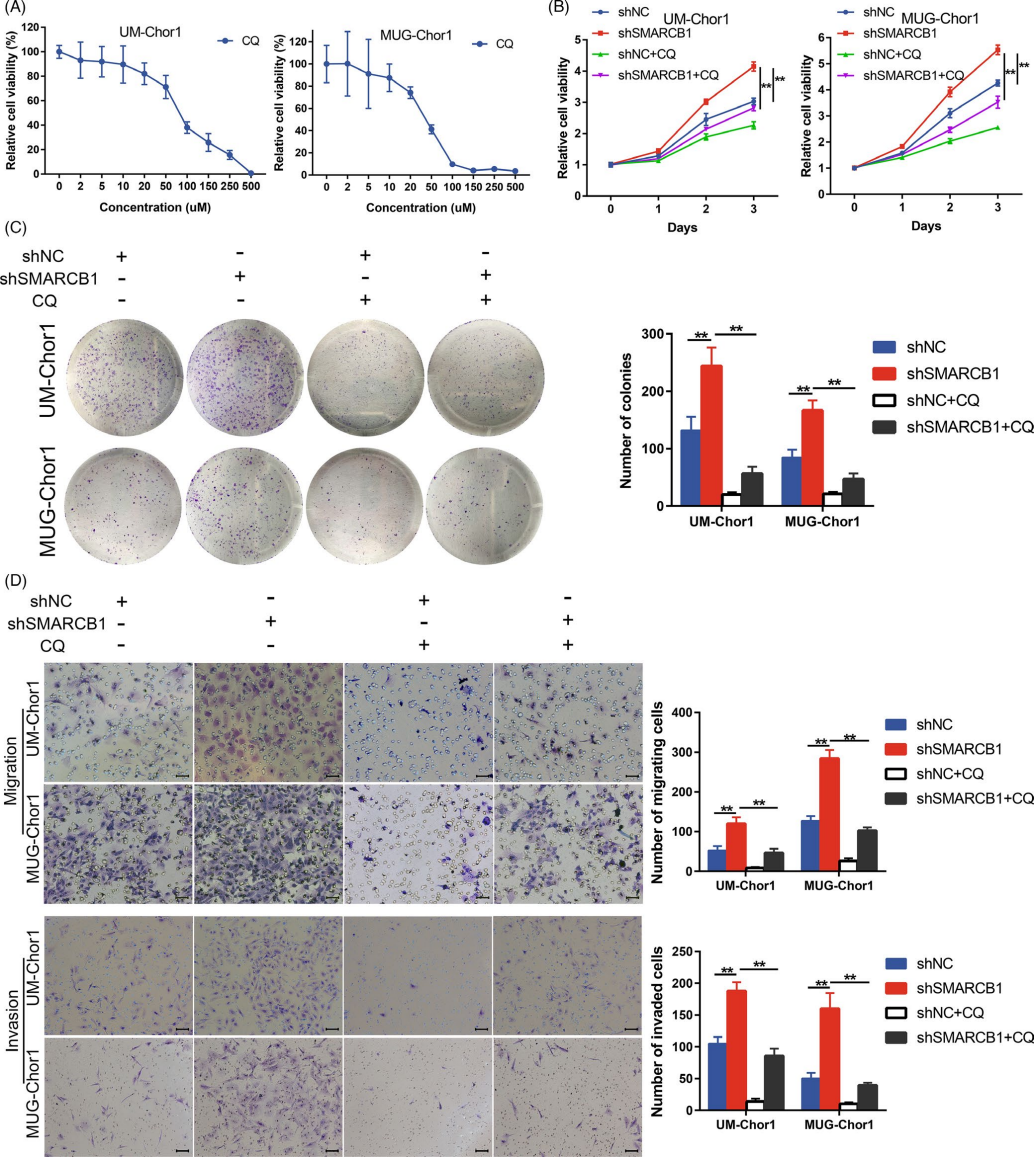
Autophagy inhibitor CQ reversed the SMARCB1 loss‐mediated malignant phenotype. (A) The dose‐dependent curves of CQ in UM‐Chor1 and MUG‐Chor1 cells. (B) CCK‐8 assay of UM‐Chor1 and MUG‐Chor1 cells with or without 20 µM CQ treatment. (C) Colony formation assay of UM‐Chor1 and MUG‐Chor1 cells with or without 20 µM CQ treatment. (D) The migration and invasion of UM‐Chor1 and MUG‐Chor1 cells with or without 20 µM CQ treatment. Scale bar, 50 µm for migration and 100 µm for invasion. One‐way ANOVA (B‐D). **p < 0.01

We also assessed the effect of autophagy inhibitor CQ in chordoma. CCK‐8 assay revealed that CQ inhibited chordoma cell proliferation in a dose‐dependent manner (Figure 5A). Additionally, CQ suppressed the proliferation and colony formation of SMARCB1 knockdown cells (Figure 5B, C). Moreover, the migration and invasion of SMARCB1 knockdown cells were significantly suppressed after CQ treatment (Figure 5D). Together, these results indicated that ATG5‐mediated autophagy was essential for the malignant phenotype of SMARCB1 knockdown chordoma cells.

### 3.5 High expression of ATG5 correlates with a poor prognosis of chordoma patients

**FIGURE 6:**
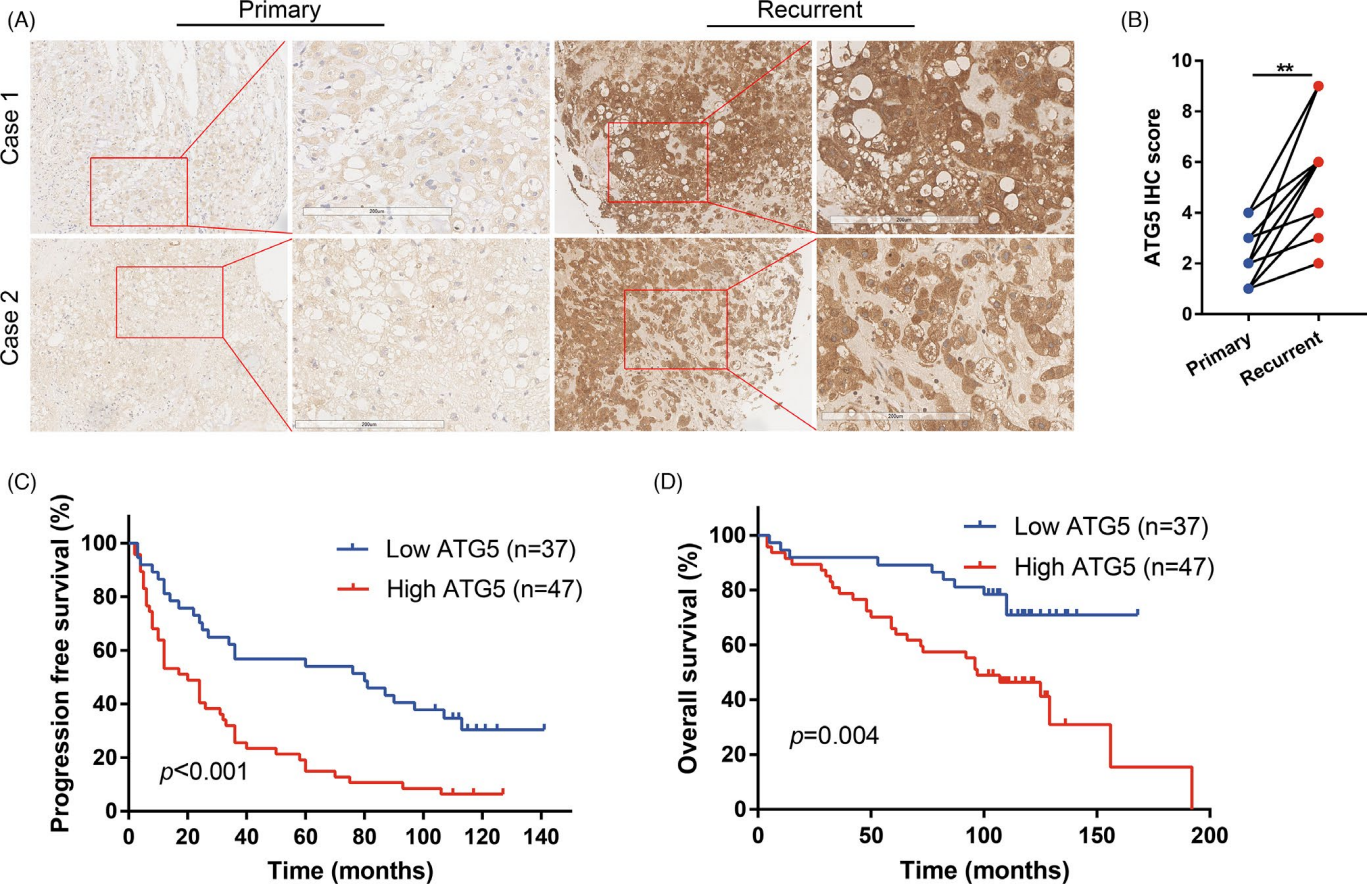
High ATG5 expression correlated with poor survival in chordoma. (A and B) IHC suggested higher ATG5 expression in recurrent chordoma compared with the corresponding primary chordoma. Scale bar, 200 µm. (C) Kaplan‐Meier curves of progression‐free survival stratified by ATG5 expression. (D) Kaplan‐Meier curves of overall survival stratified by ATG5 expression. Paired *t* test (B). Log‐rank test (C–D). **p < 0.01

We next analysed the expression of ATG5 in chordoma tissues by IHC (Figure S3). As a result, higher ATG5 expression was observed in recurrent chordoma compared with corresponding primary tumours (Figure 6A, B), suggesting an oncogenic role of ATG5 in chordoma. We then investigated potential associations between ATG5 expression and the clinical features and prognoses of the 84 chordoma patients (Table S4). Although no significant association between ATG5 and clinical features was identified, we found that high ATG5 expression was associated with shorter progression‐free survival time (median, 20 months versus 80 months) and overall survival time (median, 97 months versus >168 months) comparing with the low ATG5 expression group (Figure 6C, D). Moreover, multivariable Cox analysis identified that high ATG5 could independently predict an adverse prognosis of chordoma patients (Tables 1 and 2).

**TABLE 1:** Univariable and multivariable Cox analysis of progression‐free survival in skull base chordoma

Variables	Univariable analysis			Multivariable analysis		
	HR	95% CI	*p* value	HR	95% CI	*p* value
Age (>55/≤55 years)	1.226	0.626–2.401	0.552			
Sex (female/male)	0.980	0.607–1.581	0.933			
Tumour volume (>20/≤20 cm^3^)	2.115	1.295–3.454	0.003*	1.819	1.097–3.019	0.021*
Texture (hard or moderate/soft)	1.034	0.639–1.672	0.893			
Blood supply (abundant/poor or moderate)	1.352	0.836–2.187	0.219			
Brainstem involvement (yes/no)	1.400	0.852–2.300	0.184			
Degree of resection (non‐total/total resection)	3.252	1.545–6.847	0.002*	2.779	1.306–5.914	0.008*
ATG5 expression (high/low)	2.525	1.523–4.188	<0.001*	2.315	1.382–3.878	0.001*

Note

*p < 0.05.

**TABLE 2:** Univariable and multivariable Cox analysis of overall survival in skull base chordoma

Variables	Univariable analysis			Multivariable analysis		
	HR	95% CI	*p* value	HR	95% CI	*p* value
Age (>55/≤55 years)	0.755	0.266–2.141	0.597			
Sex (female/male)	1.170	0.617–2.221	0.631			
Tumour volume (>20/≤20 cm^3^)	1.677	0.854–3.295	0.133			
Texture (hard or moderate/soft)	1.111	0.579–2.130	0.752			
Blood supply (abundant/poor or moderate)	2.177	1.095–4.330	0.027*	1.718	0.982–3.006	0.058
Brainstem involvement (yes/no)	1.805	0.894–3.644	0.099			
Degree of resection (non‐total/total resection)	5.477	1.316–22.800	0.019*	4.998	1.195–20.901	0.028*
ATG5 expression (high/low)	2.801	1.356–5.785	0.005*	2.403	1.142–5.058	0.021*

Note

*p < 0.05.

### 3.6 SMARCB1 inhibits tumour growth and regulates autophagy in vivo

**FIGURE 7:**
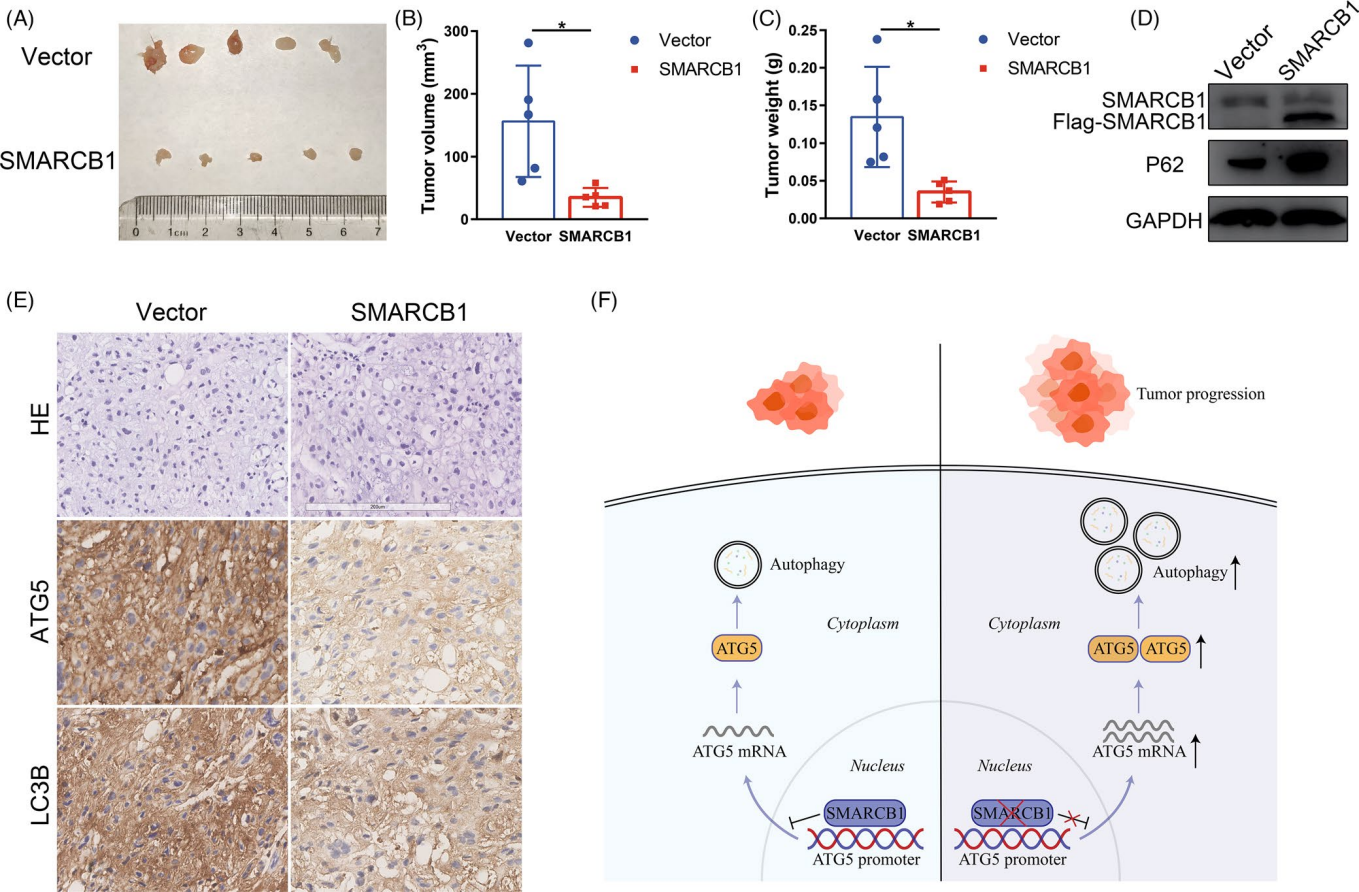
SMARCB1 inhibited chordoma progression in vivo. (A) Representative images of MUG‐Chor1 xenograft with or without SMARCB1 overexpression. (B and C) Tumour volume and weight of xenograft with or without SMARCB1 overexpression. (D) Western blot of SMARCB1 and P62 (a specific substrate for autophagy) expression of xenograft tissues. (E) Representative images of HE and IHC staining of ATG5 and LC3B in xenograft tissues. Scale bar, 200 µm. (F) Schematic model showing SMARCB1‐mediated epigenetic repression of ATG5 transcription and regulation of autophagy in chordoma. Student's *t* test (B–C). *p < 0.05

To confirm the tumour suppressive effect of SMARCB1 in chordoma in vivo, we established a xenograft model in BALB/c nude mice using MUG‐Chor1 cells with stable transfection of SMARCB1. As shown in Figure 7A–C, chordoma cells with SMARCB1 overexpression showed a delay in tumour growth compared with the vector group. Overexpression of SMARCB1 in the xenografted tissues was further confirmed (Figure 7D). Additionally, increased P62 expression and attenuated ATG5 and LC3B expression in SMARCB1‐overexpressing xenografts were observed, confirming the association between SMARCB1 and ATG5‐mediated autophagy (Figure 7D, E).

## 4 DISCUSSION

Our results confirmed the tumour suppressor role of SMARCB1 in chordoma in vitro and in vivo. Moreover, we identified ATG5 as a direct downstream target of SMARCB1 and revealed the association between autophagy and SMARCB1 in chordoma (Figure 7F). These findings may provide novel insights into the molecular mechanism by which SMARCB1 regulates cancer progression.

SMARCB1 regulates various oncogenic/tumour suppressor pathways by regulation of transcription via the form of SWI/SNF complex or interactions with transcription factors.^33^, ^34^, ^35^ Robust data have revealed that SMARCB1 functions as a tumour suppressor gene during cancer progression, although one study has reported a tumorigenic role in liver cancer.^9^, ^10^, ^15^ In chordoma, loss of SMARCB1 characterizes a novel subtype of chordoma with a dismal prognosis, namely poorly differentiated chordoma.^17^, ^18^, ^19^ Previous studies have mainly focussed on the origin of SMARCB1 loss and identified locus deletion rather than gene mutation as the potential mechanism.^17^, ^20^, ^36^ However, the downstream mechanism of SMARCB1 in chordoma remains largely unclear. Here, we confirmed the tumour suppressor role of SMARCB1 in chordoma in vitro and in vivo. Moreover, ChIP sequencing and GO analysis revealed that autophagy may be involved in the effect of SMARCB1 on chordoma cells and identified ATG5 as a novel transcriptional target of SMARCB1. Rescue experiments using ATG5 siRNA or CQ further confirmed that ATG5‐mediated autophagy played a major role in the oncogenesis of SMARCB1 loss. Recently, MYC‐p53 mediated autophagy activation was reported in SMARCB1‐deficient malignancies.^37^ Our data further supported the novel association between SMARCB1 and autophagy and suggested another molecular mechanism by which SMARCB1 mediates autophagy. Our results are in favour of the speculation that compared with SMARCB1 positive chordoma, the autophagy is enhanced in SMARCB1 negative chordoma due to the loss of SMARCB1 and subsequently transcriptional activation of ATG5, contributing to the malignant phenotype and adverse survival. We are currently collecting the clinicopathological information and tissue specimen of SMARCB1 negative chordoma (poorly differentiated chordoma) and will investigate the potential difference of autophagic level and molecular mechanism between SMARCB1 positive chordoma and SMARCB1 negative chordoma in our future work. In addition, the GO analysis suggested several potential downstream pathways of SMARCB1, such as lipid kinase activity and phospholipid metabolic process. Further studies including metabonomics and phosphorylation proteomics of chordoma cells with SMARCB1 changes or SMARCB1 positive/negative chordoma are highly recommended to comprehensively analyse the mechanism of SMARCB1 in chordoma.

Expanding attention has been focussed on autophagy because of its Janus‐faced effect on tumour progression.^38^ One previous study on chordoma reported the presence of autophagosomes and autophagolysosomes in chordoma cell lines.^39^ Additionally, a recent IHC analysis showed positive expression of autophagic markers LC3B, P62 and ATG16L1 in chordoma compared with normal notochords, which revealed the potential oncogenic role of autophagy in chordoma.^32^ Consistent with prior observations, we found positive expression of ATG5, a vital ATG during autophagy, in chordoma, especially recurrent chordoma, and identified ATG5 as a novel adverse prognostic factor of chordoma patients. Additionally, our in vitro data suggested impairments of proliferation, migration and invasion of chordoma cells after blocking autophagy by ATG5 siRNA or autophagy inhibitor CQ, revealing the tumour promotive effect of autophagy in chordoma. Our results support the assumption that autophagy is implicated in chordoma tumorigenesis,^32^ highlighting the importance of further exploring the role and regulatory mechanism of autophagy in chordoma.

Previous clinical studies have demonstrated the promising therapeutic role of autophagy inhibitors in cancer treatment alone or in combination with anti‐cancer therapies, such as chemotherapy, radiotherapy and targeted therapy.^40^, ^41^, ^42^ Our findings provide a preclinical basis for clinical trials of autophagy inhibitors, although further in vivo validation and development of other underlying autophagy‐related agents are warranted. Moreover, considering the deficiency of satisfactory chemotherapies and targeted therapies for chordoma to date,^1^, ^3^, ^6^, ^7^, ^8^, ^43^ on the basis of our preclinical data, we speculate that the use of autophagy inhibitors combined with anti‐cancer therapies may resolve this plight in chordoma.

In summary, our findings confirmed the tumour suppressor role of SMARCB1 in chordoma in vitro and in vivo. Mechanistically, SMARCB1 binds directly to the ATG5 promoter and transcriptionally regulates its expression, which subsequently regulates autophagy and the malignant phenotype of chordoma.

## CONFLICT OF INTEREST

The authors report no conflicts of interest.

## AUTHOR CONTRIBUTIONS

Mingxuan Li, Jiwei Bai and Yazhuo Zhang contributed to the conception or design of the work. Mingxuan Li, Yutao Shen, Yujia Xiong, Shuai Wang and Chuzhong Li involved in experiment, data acquisition, analysis and interpretation. All authors involved in drafting the work or revising it critically for important intellectual content, finally approved the version to be published and agreed to be accountable for all aspects of the work.

## Supporting information

Appendix S1

## Data Availability

The data that support the findings of this study are available from the corresponding author upon reasonable request.

## References

[1] Walcott BP , Nahed BV , Mohyeldin A , Coumans JV , Kahle KT , Ferreira MJ . Chordoma: current concepts, management, and future directions. Lancet Oncol. 2012;13(2):e69‐76. doi:10.1016/s1470-2045(11)70337-0 22300861

[2] Zhai Y , Bai J , Wang S , et al. Analysis of clinical factors and PDGFR‐β in predicting prognosis of patients with clival chordoma. J Neurosurg. 2018;129(6):1429‐1437. doi:10.3171/2017.6.jns17562 29303447

[3] Frezza AM , Botta L , Trama A , Dei Tos AP , Stacchiotti S . Chordoma: update on disease, epidemiology, biology and medical therapies. Curr Opin Oncol. 2019;31(2):114‐120. doi:10.1097/cco.0000000000000502 30585858

[4] Li M , Bai J , Wang S , et al. Prognostic Value of Cumulative Score Based on Preoperative Fibrinogen and Albumin Level in Skull Base Chordoma. Onco Targets Ther. 2020;13:8337‐8346. doi:10.2147/ott.s257779 32903874 PMC7445498

[5] Forander P , Bartek J Jr , Fagerlund M , et al. Multidisciplinary management of clival chordomas; long‐term clinical outcome in a single‐institution consecutive series. Acta Neurochirurgica. 2017;159(10):1857‐1868. doi:10.1007/s00701-017-3266-1 28735379 PMC5590026

[6] Stacchiotti S , Morosi C , Lo Vullo S , et al. Imatinib and everolimus in patients with progressing advanced chordoma: A phase 2 clinical study. Cancer. 2018;124(20):4056‐4063. doi:10.1002/cncr.31685 30216418

[7] Liu C , Jia Q , Wei H , et al. Apatinib in patients with advanced chordoma: a single‐arm, single‐centre, phase 2 study. Lancet Oncol. 2020;21(9):1244‐1252. doi:10.1016/s1470-2045(20)30466-6 32888455

[8] DeMaria PJ , Bilusic M , Park DM , Randomized D‐B , et al. Placebo‐Controlled Phase II Study of Yeast‐Brachyury Vaccine (GI‐6301) in Combination with Standard‐of‐Care Radiotherapy in Locally Advanced, Unresectable Chordoma. Oncologist. 2021;26(5):e847‐e858. doi:10.1002/onco.13720 33594772 PMC8100546

[9] Roberts CW , Orkin SH . The SWI/SNF complex–chromatin and cancer. Nat Rev Cancer. 2004;4(2):133‐142. doi:10.1038/nrc1273 14964309

[10] Sen P , Luo J , Hada A , et al. Loss of Snf5 Induces Formation of an Aberrant SWI/SNF Complex. Cell Rep. 2017;18(9):2135‐2147. doi:10.1016/j.celrep.2017.02.017 28249160 PMC5424545

[11] Biegel JA , Busse TM , Weissman BE . SWI/SNF chromatin remodeling complexes and cancer. Am J Med Genet C Semin Med Genet. 2014;166c(3):350–366. doi:10.1002/ajmg.c.31410 25169151 PMC4516040

[12] Jackson EM , Sievert AJ , Gai X , et al. Genomic analysis using high‐density single nucleotide polymorphism‐based oligonucleotide arrays and multiplex ligation‐dependent probe amplification provides a comprehensive analysis of INI1/SMARCB1 in malignant rhabdoid tumors. Clin Cancer Res. 2009;15(6):1923‐1930. doi:10.1158/1078-0432.ccr-08-2091 19276269 PMC2668138

[13] Christiaans I , Kenter SB , Brink HC , et al. Germline SMARCB1 mutation and somatic NF2 mutations in familial multiple meningiomas. J Med Genet. 2011;48(2):93‐97. doi:10.1136/jmg.2010.082420 20930055

[14] Kohashi K , Oda Y . Oncogenic roles of SMARCB1/INI1 and its deficient tumors. Cancer Sci. 2017;108(4):547‐552. doi:10.1111/cas.13173 28109176 PMC5406539

[15] Hong SH , Son KH , Ha SY , et al. Nucleoporin 210 Serves a Key Scaffold for SMARCB1 in Liver Cancer. Cancer Res. 2021;81(2):356‐370. doi:10.1158/0008-5472.can-20-0568 33239431

[16] Li M , Zhai Y , Bai J , et al. SNF5 as a prognostic factor in skull base chordoma. J Neurooncol. 2018;137(1):139‐146. doi:10.1007/s11060-017-2706-3 29222701

[17] Hasselblatt M , Thomas C , Hovestadt V , et al. Poorly differentiated chordoma with SMARCB1/INI1 loss: a distinct molecular entity with dismal prognosis. Acta Neuropathol. 2016;132(1):149‐151. doi:10.1007/s00401-016-1574-9 27067307

[18] Mobley BC , McKenney JK , Bangs CD , et al. Loss of SMARCB1/INI1 expression in poorly differentiated chordomas. Acta Neuropathol. 2010;120(6):745‐753. doi:10.1007/s00401-010-0767-x 21057957

[19] Antonelli M , Raso A , Mascelli S , et al. SMARCB1/INI1 Involvement in Pediatric Chordoma: A Mutational and Immunohistochemical Analysis. Am J Surg Pathol. 2017;41(1):56‐61. doi:10.1097/pas.0000000000000741 27635948

[20] Yeter HG , Kosemehmetoglu K , Soylemezoglu F . Poorly differentiated chordoma: review of 53 cases. Apmis. 2019;127(9):607‐615. doi:10.1111/apm.12978 31243811

[21] Mizushima N , Komatsu M . Autophagy: renovation of cells and tissues. Cell. 2011;147(4):728‐741. doi:10.1016/j.cell.2011.10.026 22078875

[22] Mizushima N , Levine B . Autophagy in mammalian development and differentiation. Nat Cell Biol. 2010;12(9):823‐830. doi:10.1038/ncb0910-823 20811354 PMC3127249

[23] Sui X , Chen R , Wang Z , et al. Autophagy and chemotherapy resistance: a promising therapeutic target for cancer treatment. Cell Death Dis. 2013;4(10):e838. doi:10.1038/cddis.2013.350 24113172 PMC3824660

[24] Levy JMM , Towers CG , Thorburn A . Targeting autophagy in cancer. Nat Rev Cancer. 2017;17(9):528‐542. doi:10.1038/nrc.2017.53 28751651 PMC5975367

[25] Degenhardt K , Mathew R , Beaudoin B , et al. Autophagy promotes tumor cell survival and restricts necrosis, inflammation, and tumorigenesis. Cancer Cell. 2006;10(1):51‐64. doi:10.1016/j.ccr.2006.06.001 16843265 PMC2857533

[26] Le Bars R , Marion J , Le Borgne R , Satiat‐Jeunemaitre B , Bianchi MW . ATG5 defines a phagophore domain connected to the endoplasmic reticulum during autophagosome formation in plants. Nature Commun. 2014;5(1):4121. doi:10.1038/ncomms5121 24947672

[27] Yousefi S , Perozzo R , Schmid I , et al. Calpain‐mediated cleavage of Atg5 switches autophagy to apoptosis. Nat Cell Biol. 2006;8(10):1124‐1132. doi:10.1038/ncb1482 16998475

[28] Yang PW , Hsieh MS , Chang YH , Huang PM , Lee JM . Genetic polymorphisms of ATG5 predict survival and recurrence in patients with early‐stage esophageal squamous cell carcinoma. Oncotarget. 2017;8(53):91494‐91504. doi:10.18632/oncotarget.20793 29207660 PMC5710940

[29] Li M , Bai J , Wang S , et al. Clinical Implication of Systemic Immune‐Inflammation Index and Prognostic Nutritional Index in Skull Base Chordoma Patients. Front Oncol. 2021;11:548325. doi:10.3389/fonc.2021.548325 33718126 PMC7947628

[30] Li M , Bai J , Wang S , et al. Mean platelet volume and platelet distribution width serve as prognostic biomarkers in skull base chordoma: a retrospective study. BMC Cancer. 2020;20(1):988. doi:10.1186/s12885-020-07497-7 33046024 PMC7552483

[31] Liu L , Wang T , Yang X , et al. MTNR1B loss promotes chordoma recurrence by abrogating melatonin‐mediated β‐catenin signaling repression. J Pineal Res. 2019;67(2):e12588. doi:10.1111/jpi.12588 31140197

[32] Karpathiou G , Dridi M , Krebs‐Drouot L , et al. Autophagic Markers in Chordomas: Immunohistochemical Analysis and Comparison with the Immune Microenvironment of Chordoma Tissues. Cancers. 2021;13(9):2169. doi:10.3390/cancers13092169 33946484 PMC8124629

[33] Wang X , Lee RS , Alver BH , et al. SMARCB1‐mediated SWI/SNF complex function is essential for enhancer regulation. Nat Genet. 2017;49(2):289‐295. doi:10.1038/ng.3746 27941797 PMC5285474

[34] Cheng SW , Davies KP , Yung E , Beltran RJ , Yu J , Kalpana GV . c‐MYC interacts with INI1/hSNF5 and requires the SWI/SNF complex for transactivation function. Nat Genet. 1999;22(1):102‐105. doi:10.1038/8811 10319872

[35] Weissmiller AM , Wang J , Lorey SL , et al. Inhibition of MYC by the SMARCB1 tumor suppressor. Nature Commun. 2019;10(1):2014. doi:10.1038/s41467-019-10022-5 31043611 PMC6494882

[36] Bai J , Shi J , Li C , et al. Whole genome sequencing of skull‐base chordoma reveals genomic alterations associated with recurrence and chordoma‐specific survival. Nat Commun. 2021;12(1):757. doi:10.1038/s41467-021-21026-5 33536423 PMC7859411

[37] Carugo A , Minelli R , Sapio L , et al. p53 Is a Master Regulator of Proteostasis in SMARCB1‐Deficient Malignant Rhabdoid Tumors. Cancer Cell. 2019;35(2):204‐220.e9. doi:10.1016/j.ccell.2019.01.006 30753823 PMC7876656

[38] White E , Mehnert JM , Chan CS . Autophagy, metabolism, and cancer. Clin Cancer Res. 2015;21(22):5037‐5046. doi:10.1158/1078-0432.ccr-15-0490 26567363 PMC4646728

[39] Kolb‐Lenz D , Fuchs R , Lohberger B , et al. Characterization of the endolysosomal system in human chordoma cell lines: is there a role of lysosomes in chemoresistance of this rare bone tumor? Histochem Cell Biol. 2018;150(1):83‐92. doi:10.1007/s00418-018-1673-x 29725750

[40] Horne GA , Stobo J , Kelly C , et al. A randomised phase II trial of hydroxychloroquine and imatinib versus imatinib alone for patients with chronic myeloid leukaemia in major cytogenetic response with residual disease. Leukemia. 2020;34(7):1775‐1786. doi:10.1038/s41375-019-0700-9 31925317 PMC7224085

[41] Mulcahy Levy JM , Thorburn A . Autophagy in cancer: moving from understanding mechanism to improving therapy responses in patients. Cell Death Differ. 2020;27(3):843‐857. doi:10.1038/s41418-019-0474-7 31836831 PMC7206017

[42] Compter I , Eekers DBP , Hoeben A , et al. Chloroquine combined with concurrent radiotherapy and temozolomide for newly diagnosed glioblastoma: a phase IB trial. Autophagy. 2020;1‐9. Online ahead of print. 10.1080/15548627.2020.1816343 PMC8496728 32866424

[43] Meng T , Jin J , Jiang C , et al. Molecular targeted therapy in the treatment of chordoma: a systematic review. Front Oncol. 2019;9:30. doi:10.3389/fonc.2019.00030 30775316 PMC6367227

