# Supplementary material for: Loss of SMARCB1 promotes autophagy and facilitates tumour progression in chordoma by transcriptionally activating ATG5
Source: Cell Prolif. 2021 Oct 20;54(12):e13136. doi: 10.1111/cpr.13136 (PMC8666275; doi:10.1111/cpr.13136)
Supplement: Supplementary file 1 — Appendix S1 [file CPR-54-e13136-s001.docx]

**Supplementary Table 1** Sequence of lentivirus vectors and siRNA

| Name | Sequence (5’-3’) |
| --- | --- |
| shNC lentivirus | TTCTCCGAACGTGTCACGT |
| shSMARCB1 lentivirus | GCAACGATGAGAAGTACAA |
| siATG5 | TGACGTTGGTAACTGACAA |

**Supplementary Table 2** Primers used for PCR

| Name |  | Sequence (5’-3’) |
| --- | --- | --- |
| SMARCB1 | Forward | ATCACGGATACACGACTCTAGC |
|  | Reverse | CACGGCATCTAAGTGGTGGG |
| GAPDH | Forward | GGAGCGAGATCCCTCCAAAAT |
|  | Reverse | GGCTGTTGTCATACTTCTCATGG |
| ATG5 | Forward | AGAAGCTGTTTCGTCCTGTGG |
|  | Reverse | AGGTGTTTCCAACATTGGCTC |
| ATG7 | Forward | CAGTTTGCCCCTTTTAGTAGTGC |
|  | Reverse | CCAGCCGATACTCGTTCAGC |
| Beclin | Forward | CCATGCAGGTGAGCTTCGT |
|  | Reverse | GAATCTGCGAGAGACACCATC |
| ULK1 | Forward | GGCAAGTTCGAGTTCTCCCG |
|  | Reverse | CGACCTCCAAATCGTGCTTCT |
| AMBRA1 | Forward | CTGGTAGAAGATAAAACCCGGTG |
|  | Reverse | AGGTAGAGCGTGGACTATCCG |
| ATG5-ChIP-primers | Forward | GCCGGTTGTATTCGCTGG |
|  | Reverse | AGCAGGACTCCAGGAAGC |

**Supplementary Table 3** Probes for EMSA

| Name | Sequence |
| --- | --- |
| ATG5-EMSA-probe 1 | GGTGCGCTGCCAGGGCTCCGCAGCGTTGCCGGTTGTATTCGCTGGATACC |
| ATG5-EMSA-probe 2 | AGAGGGCGGAAGTGCAGCAGGGTTCAGCTCCGACCTCCGCGCCGGTGCTT |
| ATG5-EMSA-probe 3 | TTTGCGGCTGCGCGGGCTTCCTGGAGTCCTGCTACCGCGTCCCCGCAGGACA |
| ATG5-EMSA-probe 4 | GTGTGTCAGGCGGGCAGCTTGCCCCGCCGCCCCACCGGAGCGCGGAATCTGG |
| ATG5-EMSA-probe 5 | GCGTCCCCACCAGTGCGGGGAGCCGGAAGGAGGAGCCATAGCTTGGAGTAGG |

**Supplementary Table 4** Clinicopathological features of 84 skull base chordoma patients and their correlations with ATG5 expression

| Variables | Number of patients | | | *P-*value |
| --- | --- | --- | --- | --- |
|  | Total | High ATG5 | Low ATG5 |  |
| Age |  |  |  | 0.524 |
| ≤55 | 73 | 42 | 31 |  |
| >55 | 11 | 5 | 6 |  |
| Gender |  |  |  | 0.949 |
| Male | 48 | 27 | 21 |  |
| Female | 36 | 20 | 16 |  |
| Tumor volume |  |  |  | 0.150 |
| ≤20 cm^3^ | 38 | 18 | 20 |  |
| >20 cm^3^ | 46 | 29 | 17 |  |
| Blood supply |  |  |  | 0.232 |
| Abundant | 47 | 29 | 18 |  |
| Others (poor or moderate) | 37 | 18 | 19 |  |
| Texture |  |  |  | 0.365 |
| Soft | 34 | 17 | 17 |  |
| Others (hard or moderate) | 50 | 30 | 20 |  |
| Brainstem involvement |  |  |  | 0.875 |
| No | 31 | 17 | 14 |  |
| Yes | 53 | 30 | 23 |  |

**
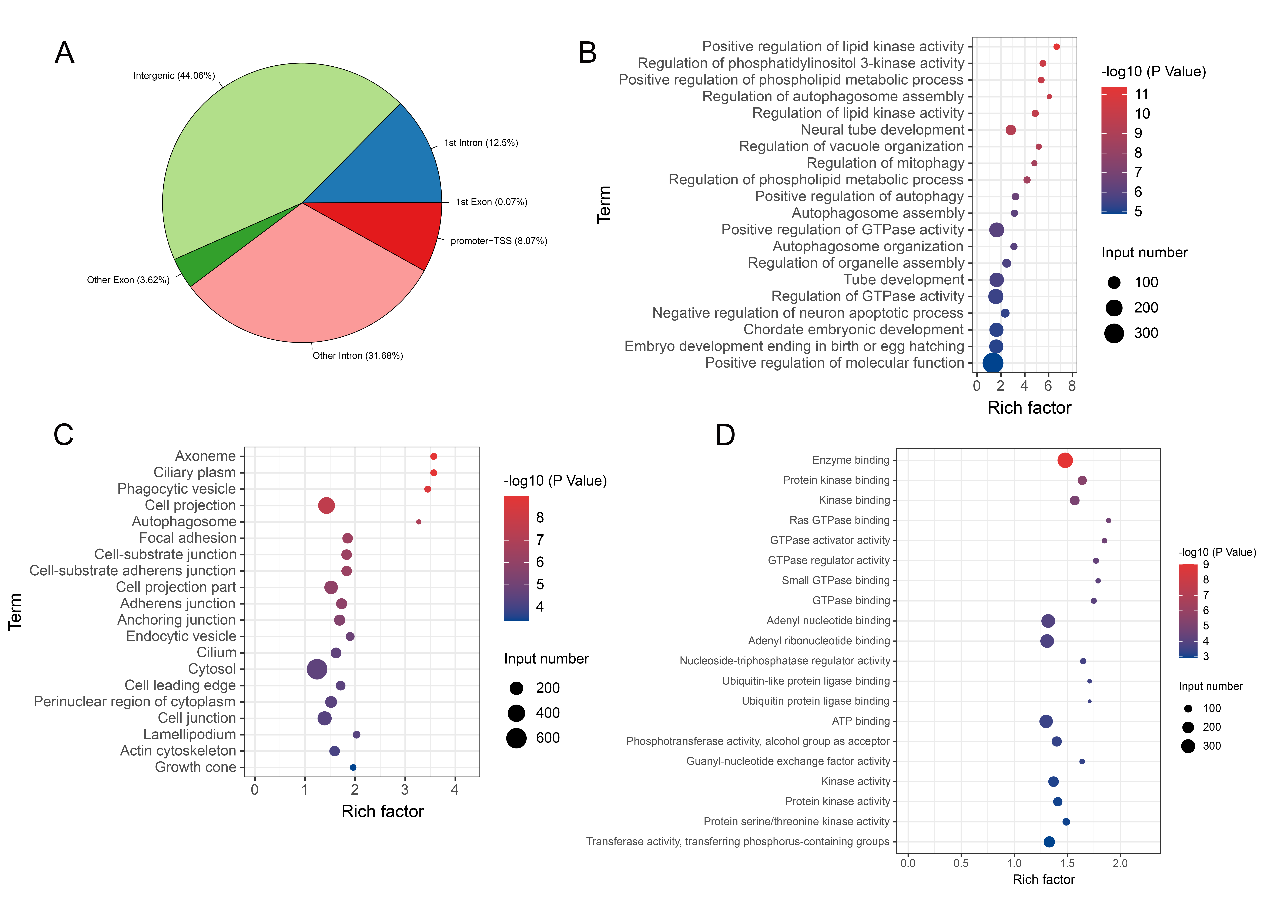
**

**Supplementary Figure 1** ChIP sequence results of SMARCB1 in UM-Chor1 cells. **(A)** Distribution of peaks in functional regions of the genome. Biological process **(B)**, cellular component **(C)**, and molecular function **(D)** of Gene Ontology of peaks-related genes.

**
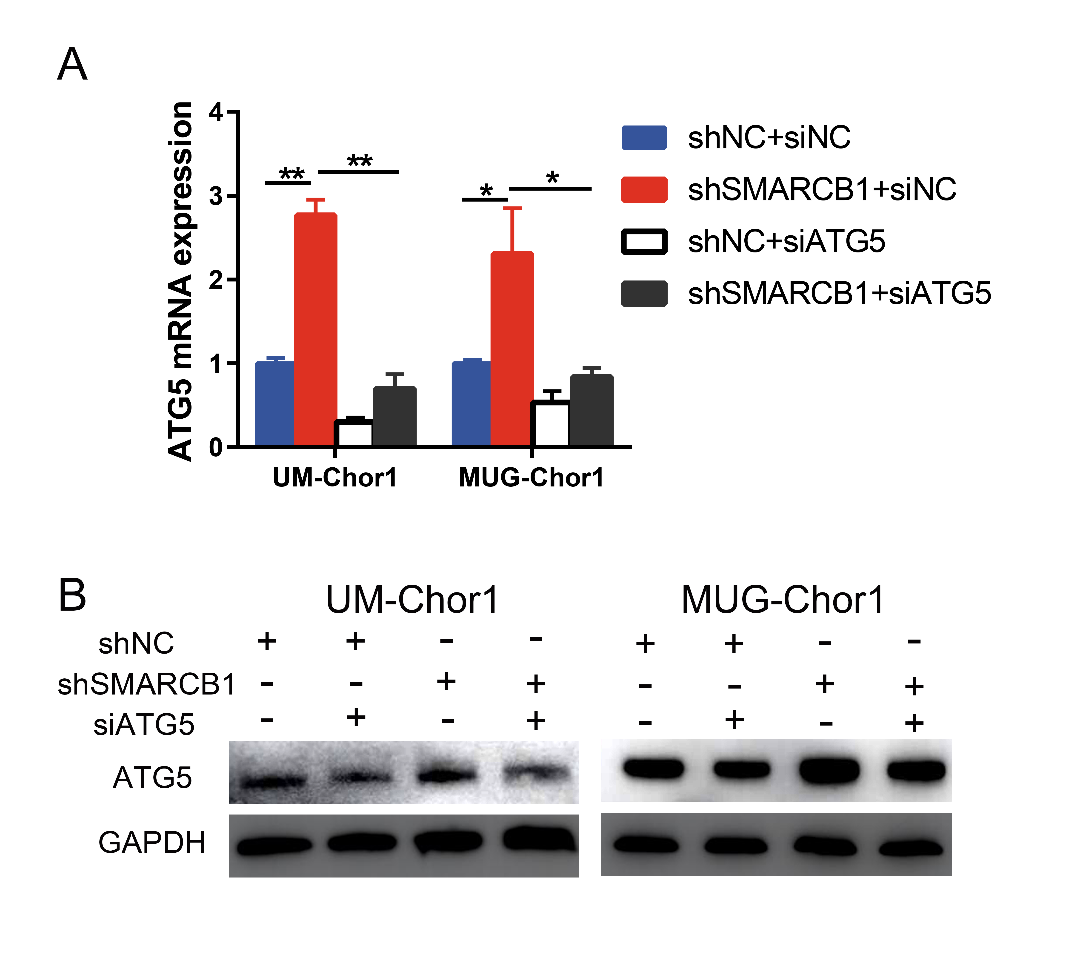
**

**Supplementary Figure 2** The knockdown efficiency of ATG5 siRNA was validated in chordoma cells. **(A)** ATG5 mRNA expression in UM-Chor1 and MUG-Chor1 cells with or without ATG5 siRNA. **(B)** ATG5 protein expression in UM-Chor1 and MUG-Chor1 cells with or without ATG5 siRNA.


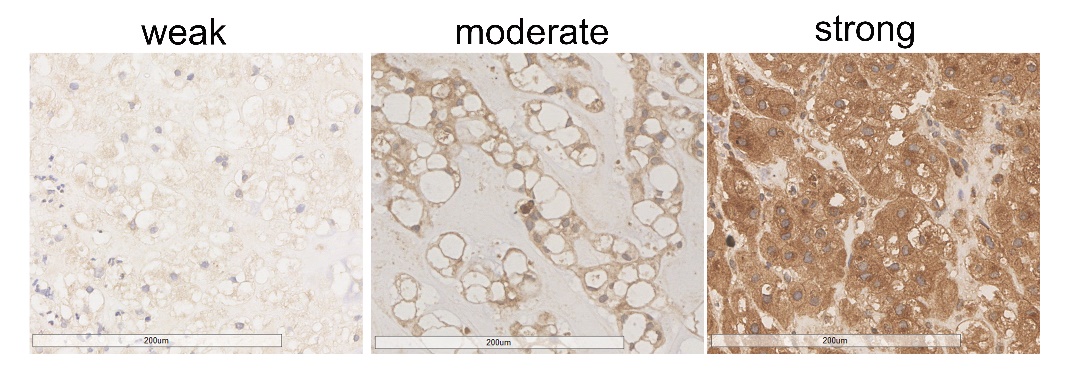


**Supplementary Figure 3** Representative images of weak, moderate, and strong IHC staining of ATG5 in chordoma tissues. Scale bar, 200 µm.
